# Is Penguin Circovirus Circulating Only in the Antarctic Circle? Lack of Viral Detection in Namibia

**DOI:** 10.3390/ani13091449

**Published:** 2023-04-24

**Authors:** Laura C. Roberts, Umberto Molini, Lauren M. Coetzee, Siegfried Khaiseb, Jean-Paul Roux, Jessica Kemper, David G. Roberts, Katrin Ludynia, Marcus Doherr, Darrell Abernethy, Giovanni Franzo

**Affiliations:** 1 Department of Production Animal Studies, Faculty of Veterinary Science, University of Pretoria, Pretoria 0110, South Africa; 2 Centre for Veterinary Wildlife Research, Faculty of Veterinary Science, University of Pretoria, Pretoria 0110, South Africa; 3 School of Veterinary Medicine, Faculty of Health Sciences and Veterinary Medicine, Neudamm Campus, University of Namibia, Private Bag, Windhoek 13301, Namibia; 4 Central Veterinary Laboratory (CVL), 24 Goethe Street, Private Bag, Windhoek 18137, Namibia; 5 African Penguin Conservation Project, Lüderitz 23016, Namibia; 6 Southern African Foundation for the Conservation of Coastal Birds (SANCCOB), Cape Town 7441, South Africa; 7 Department of Biodiversity & Conservation Biology, University of the Western Cape, Bellville 7535, South Africa; 8 Institute for Veterinary Epidemiology and Biostatistics, Department of Veterinary Medicine, University of Freie, Königsweg 67, 14163 Berlin, Germany; 9 Aberystwyth School of Veterinary Science, Department of Life Sciences, Aberystwyth University, Aberystwyth SY23 3DA, UK; 10 Department of Animal Medicine, Production and Health, University of Padova, Legnaro, 35020 Padova, Italy

**Keywords:** penguin circovirus (PenCV), Namibia, penguin, molecular epidemiology

## Abstract

**Simple Summary:**

The number of circovirus species is continuously expanding thanks to improved diagnostic and sequencing technologies. Recently, a new circovirus (penguin circovirus (PenCV)) was identified in the guano and cloacal samples collected from Adélie penguins (*Pygoscelis adeliae*) and chinstrap penguins (*Pygoscelis antarcticus*) in Antarctica, and a potential association with disease was proposed. The present study investigates the occurrence of PenCV infection in Namibian African penguin (*Spheniscus demersus*) colonies. No evidence of viral circulation was observed, suggesting that PenCV distribution could be limited to Antarctica or to particular penguin species.

**Abstract:**

The known host range of circoviruses is continuously expanding because of more intensive diagnostic activities and advanced sequencing tools. Recently, a new circovirus (penguin circovirus (PenCV)) was identified in the guano and cloacal samples collected from Adélie penguins (*Pygoscelis adeliae*) and chinstrap penguins (*Pygoscelis antarcticus*) in Antarctica. Although the virus was detected in several asymptomatic subjects, a potential association with feather disease was speculated. To investigate the occurrence and implications of PenCV in other penguin species located outside of Antarctica, a broad survey was undertaken in African penguins (*Spheniscus demersus*) on two islands off the southern Namibian coast. For this purpose, specific molecular biology assays were developed and validated. None of the 151 blood samples tested positive for PenCV. Several reasons could explain the lack of PenCV positive samples. African penguins and *Pygoscelis* species are separated by approximately 6000 km, so there is almost no opportunity for transmission. Similarly, host susceptibility to PenCV might be penguin genus-specific. Overall, the present study found no evidence of PenCV in African penguin colonies in Namibia. Further dedicated studies are required to assess the relevance of PenCV among different penguin species.

## 1. Introduction

The genus *Circovirus* includes non-enveloped viruses characterized by an ambisense single-stranded DNA (ssDNA) circular genome of approximately 2 kb, comprising two main open reading frames (ORFs). ORF1 encodes the replicase protein (Rep) involved in genome replication, while ORF2 encodes the Cap protein, the only constituent of the viral capsid [1,2]. Discovered for the first time in the 1970s as a contaminant of porcine cell lines, circoviruses have long been associated with bird infections and associated diseases (e.g., psittacine beak and feather disease virus (BFDV), pigeon circovirus (PiCV), and goose circovirus (GoCV)), including immunodeficiency syndromes [3]. An important change in our understanding of these viruses occurred with the emergence of Porcine circovirus 2 (PCV-2) and its devastating impact on swine farming, which has catalyzed the interest and research around this viral genus [4]. To date, the genus includes 49 species officially recognized by the International Committee on Taxonomy of Viruses (ICTV) (accessed 22 February 2023). Although most have limited or no impact on animal health, and for some, even the host is uncertain, others are considered a potential threat to livestock and companion animals [1]. There is a growing concern regarding their impact on wildlife health and the associated implications for endangered species conservation [5,6,7]. African penguins (*Spheniscus demersus*) are one of these endangered species, with the population having declined by approximately 65% since 1989 [8]. Recently, a new species of circovirus, penguin circovirus (PenCV), has been detected in an Adélie penguin (*Pygoscelis adeliae*) with a feather-loss disorder during the 2018–2019 breeding season at Cape Crozier, Ross Island, Antarctica and from retrospective samples collected from healthy subjects at the same location [9]. Thereafter, the same virus was identified in Port Charcot (PC), Booth Island, Southwest Antarctic Peninsula from samples taken during the 2015−2016 breeding season from one Adélie penguin and one chinstrap penguin (*Pygoscelis antarcticus*) [10]. Previous studies reported the occurrence of feather diseases in emperor penguins (*Aptenodytes forsteri*) at Cape Washington, Antartica in 1996, African penguins in South Africa in 2006 and 2008, and Magellanic penguins (*Spheniscus magellanicus*) in four colonies in Argentina [9,11]. Adélie penguins at Cape Royds and at Esperanza Bay, Antarctica were observed with similar clinical manifestations in 2011–2012 and in 2013–2014, respectively [12]. Despite viral etiology being hypothesized, none of the lesions were associated with viruses known to infect penguins (see [13]). The potential role of the newly discovered PenCV was suggested because of the analogy with other bird-infecting circoviruses causing feather abnormalities. Biomolecular assays performed on cloacal swabs collected from 25 healthy penguins in the same region highlighted a relatively high infection frequency (12%) [9]. However, the limited sample size and the multifactorial nature of circovirus diseases prevented any definitive conclusion on the PenCV pathogenicity and virulence. Because of the relevance that certain circoviruses had on animal welfare and health, and the potential implications in wildlife conservation, a precise understanding of PenCV distribution, association with clinical signs, and impact of wild penguin population dynamics would be of great benefit.

To improve knowledge on these topics, sera from African Penguins were tested for PenCV.

## 2. Materials and Methods

### 2.1. Sampling

**Figure 1:**
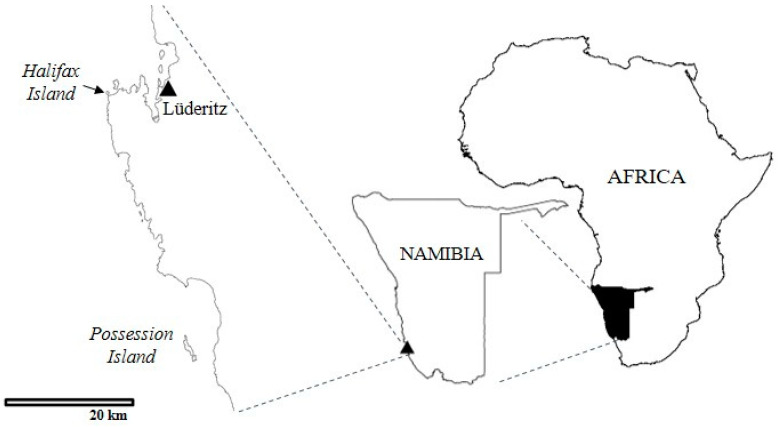
Map reporting the location of the sampling sites.

The samples were collected on Halifax and Possession Islands, off the southern coast of Namibia (Figure 1).

The islands are adjacent to the Namib Desert and are the southernmost of four islands where the majority of the Namibian African penguins have bred historically. The population in Namibia was estimated in 2017 to be approximately 5500 breeding pairs, with approximately 27% on Halifax and 17% on Possession Island (Ministry of Fisheries and Marine Resources unpublished data). On Halifax Island, the penguins breed mostly in open nests, in five discrete, densely populated colonies. The island is approximately 100 m from the mainland at the closest point and has a diameter of approximately 400 × 440 m. Possession Island is approximately 40 km south of Halifax and approximately 2.5 km from the mainland. The penguin nests there are more widely dispersed, though there are aggregations in some places, and there are more earth burrows, as well as nests under sparse low bushes. Possession Island, which is approximately 3.4 km long and 650 m at its widest, is much larger than Halifax Island. Penguins breed throughout the year in Namibia, with breeding activities generally peaking during austral summer. Samples were taken as part of a larger study to assess African penguin health (Africanpenguinhealth.co.za). Sample size was calculated using a modified binomial approximation analysis for disease freedom at a prevalence lower than 5%, based on the estimated adult-plumaged populations of 4700 and 3000 individuals on Halifax and Possession Islands, respectively, from the 2017 census, and assuming a conservative test sensitivity and specificity of 96% [14].

Birds in adult plumage (older than approximately 18 months) were selected and, to minimize the stress of capture, only those standing in the colonies on Halifax or in nests and/or defending chicks were sampled. Samples were distributed between colonies on Halifax Island, proportional to size, and across Possession Island. On Halifax, it was only possible to sample birds at the periphery of the colonies to avoid excessive disturbance, and, on both islands, birds on eggs and with small chicks were excluded, as well as those undergoing molt. A total of 151 serum samples were collected, including 102 on Halifax Island in March 2021 and 49 on Possession Island in April 2022.

Blood samples were collected from the jugular vein using a sterile 20 mL syringe and a 21G 1.5″ needle and transferred to either silica-coated clot activator serum tubes or gel serum separator tubes. The tubes were kept cool in an insulated bag with an ice pack, or in a refrigerator, until processing. Serum was obtained by centrifugation of serum tubes at 1690× *g* for 15 min and it was then refrigerated before it was sent to the Central Veterinary Laboratory (CVL; Windhoek, Namibia) for freezing at −20 °C, and later, PenCV investigation.

### 2.2. PenCV Molecular Testing

A qPCR assay was validated for PenCV detection by designing primer pairs and probes (see below) using Primer3Plus, based on the available GenBank sequence (i.e., MN164704.1) [9,10]. Since no reference positive control was available, the complete genome of PenCV was chemically synthesized (GenScript Biotech, Piscataway, NJ, USA) and cloned in a pUC57-Kan plasmid. The analytic sensitivity of the assay was evaluated by performing a ten-fold serial dilution of the plasmid that was tested in 10 independent runs. The last dilution detected in 90% of the replicates was considered the limit of detection (LoD) of the assay (i.e., 10 copies/µL). Analytic specificity was assessed by testing a panel (i.e., BFDV; CaninceCV; PCV-1,-2,-3 and 4) of known circovirus infecting both birds and mammalian hosts. DNA was extracted from penguin serum samples using a High Pure Viral Nucleic Acid Kit (Hoffman, Switzerland) with an elution volume of 100 μL, following the manufacturer’s instructions. All samples were screened using a real-time PCR (qPCR) assay with specific primers and probe as follows: qPenCV_F (ATGTGGCTCTTGAGTTCCCG′), qPenCV_R (AACGTGACTTCCCACATCCC), and qPenCV_Probe (FAM-CGCTCTTCTCATTGGACGCGCGCCT-BHQ1). Briefly, qPCR was performed on a C1000 Bio-Rad thermocycler (Bio-Rad, Hercules, CA, USA) with PrecisionPLUS qPCR Mastermix (Genesig Primerdesign Ltd., Camberley, UK). The cycling conditions were 95 °C for 10 min, followed by 45 cycles of 95 °C for 15 s, and 60 °C for 1 min. The fluorescence signal was acquired at the end of each cycle extension phase. Positive (i.e., 100 copies/µL of PenCV plasmid) and negative (i.e., ultrapure, molecular-grade water) controls were included in each run. An exogenous internal control (IC), described in Franzo et al., 2018 [15], was added to each sample before extraction (1000 copies/extraction) and simultaneously tested in the same reaction. Briefly, a segment of enhanced green fluorescent protein (EGFP) gene was amplified and inserted in a standard cloning vector. Primers and probes specific for IC were selected according to Hoffmann et al., 2006 [16] as follows: EGFP-1-F (GACCACTACCAGCAGAACAC), EGFP-2-R (GAACTCCAGCAGGACCATG), and EGFP-Probe (HEX-AGCACCCAGTCCGCCCTGAGCA-BHQ-2).

To differentiate PenCV from IC amplification during the multiplex qPCR, the IC probe was marked with HEX fluorophore.

## 3. Results

The only clinical abnormality recorded, related to feathers, was one bird with some feathers missing above an eye. The internal control was successfully amplified in all samples; however, none of the considered samples tested PenCV positive.

## 4. Discussion

Based on the obtained sample size and diagnostic test performances, it is reasonable to conclude that PenCV, if present, had a prevalence of 5% or less. Because of the successful amplification of all positive and IC controls, false negative and failure in extraction or inhibitors presence can be excluded. There are several different explanations for the apparent absence of PenCV. If the virus originated in Antarctica, the geographical distance between the Namibian colonies and the Antarctic colony where PenCV was reported, and hence, a lack of direct contact between the two penguin species, could explain the lack of viral spread. Alternatively, host susceptibility to infection by PenCV could be genus- or species-specific. Although circovirus infection is characterized by a certain degree of plasticity in host tropism [17], the specific host determinants have never been established. The evidence that PenCV was detected in Adélie and Chinstrap penguins and feather abnormalities were previously reported in penguins belonging to different genera and from distant geographic areas could weaken these hypotheses. However, there is no robust evidence that PenCV was involved in all (or any) of the cases and no conclusion can thus be drawn. Additionally, it must be highlighted that serum samples were tested in the present study. There is evidence from other studies [18,19] that avian circoviruses can be detected in sera, but all of the previous positive PenCV samples were guano or cloacal samples. Therefore, food-related contamination, rather than a true infection, was possible, although the higher genetic similarity with gull (*Larus* spp.) circoviruses (GuCV) would suggest a common origin of the two viruses, and thus, a similar host range. A viral tropism for the intestinal tract, favoring higher titers in the guano than in blood cannot be excluded and would deserve further investigation. Finally, a low prevalence, below the detection level of the present study, cannot be excluded, although it would be in contrast with previously reported prevalence [9,10].

## 5. Conclusions

Overall, the present study results report no detectable evidence of PenCV in penguin colonies in Namibia. There is no clear explanation as to why the African penguin samples were negative, so an open mind regarding these results should be kept and its existence cannot be definitively ruled out. Any future African penguin population health surveys in Namibia should include testing for this virus in their protocols.

Moreover, further studies on PenCV in other penguin and bird species are required to assess the impact and relevance of this newly described virus.

## Data Availability

Not applicable.
